# Quantification of coronary vessel wall thickness using a flexible time-resolved golden angle dual-inversion recovery acquisition for facilitated sequence timing at 3T

**DOI:** 10.1186/1532-429X-18-S1-W35

**Published:** 2016-01-27

**Authors:** Giulia Ginami, Jérôme Yerly, Pier Giorgio Masci, Matthias Stuber

**Affiliations:** 1 CIBM/CHUV/UNIL Lausanne, Lausanne, Switzerland; 2 Radiology, Division of Cardiology and Cardiac MR Centre, CHUV, Lausanne, Switzerland

## Background

Positive vessel wall remodeling is an early marker of coronary artery diseases. Its early detection could improve prognosis of adverse cardiovascular events. Black blood MRI based on double inversion recovery (DIR) (1) represents a non-invasive technique for the visualization of the coronary vessel wall. However, the need of collecting imaging data at the moment of both optimal blood signal nulling (2) and minimal myocardial motion, still limits the use of the technique in clinic. In order to alleviate difficulties in sequence planning, we propose a continuous acquisition scheme throughout a prolonged acquisition window. Combined with golden angle radial acquisition and k-t sparse SENSE (3), this enables a fully flexible a posteriori selection of imaging parameters.

## Methods

**Figure 1:**
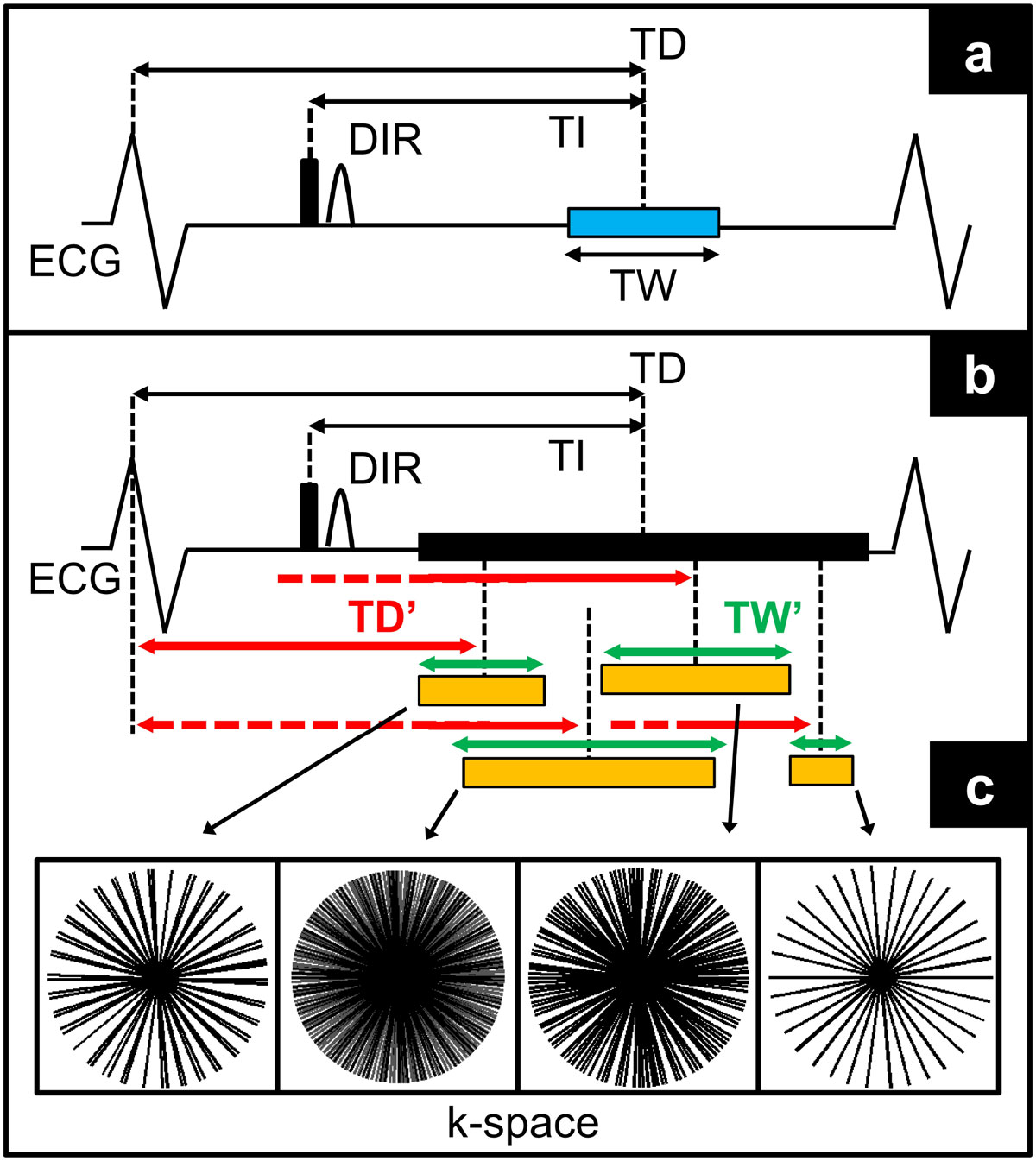
**Long-window acquisition scheme**. A conventional single-frame DIR acquisition is shown in a; the trigger delay (TD) is selected in order to place the acquisition window (blue box) in correspondence to the period of minimal cardiac motion. Simultaneously, a heart-dependent inversion time (TI) is prescribed in order to ensure data acquisition at the moment of optimal blood signal nulling. In b, the long window acquisition is shown. The acquisition window is prolonged and, during post-processing, sub-divided into multiple frames (orange boxes). Because of the golden angle trajectory, frames position (TD', red arrows) within the cardiac cycle, as well as frames duration (green arrows), can be freely selected. In order to compensate for high undersampling in shorter frames (c), image reconstruction is performed with k-t sparse SENSE.

**Figure 2:**
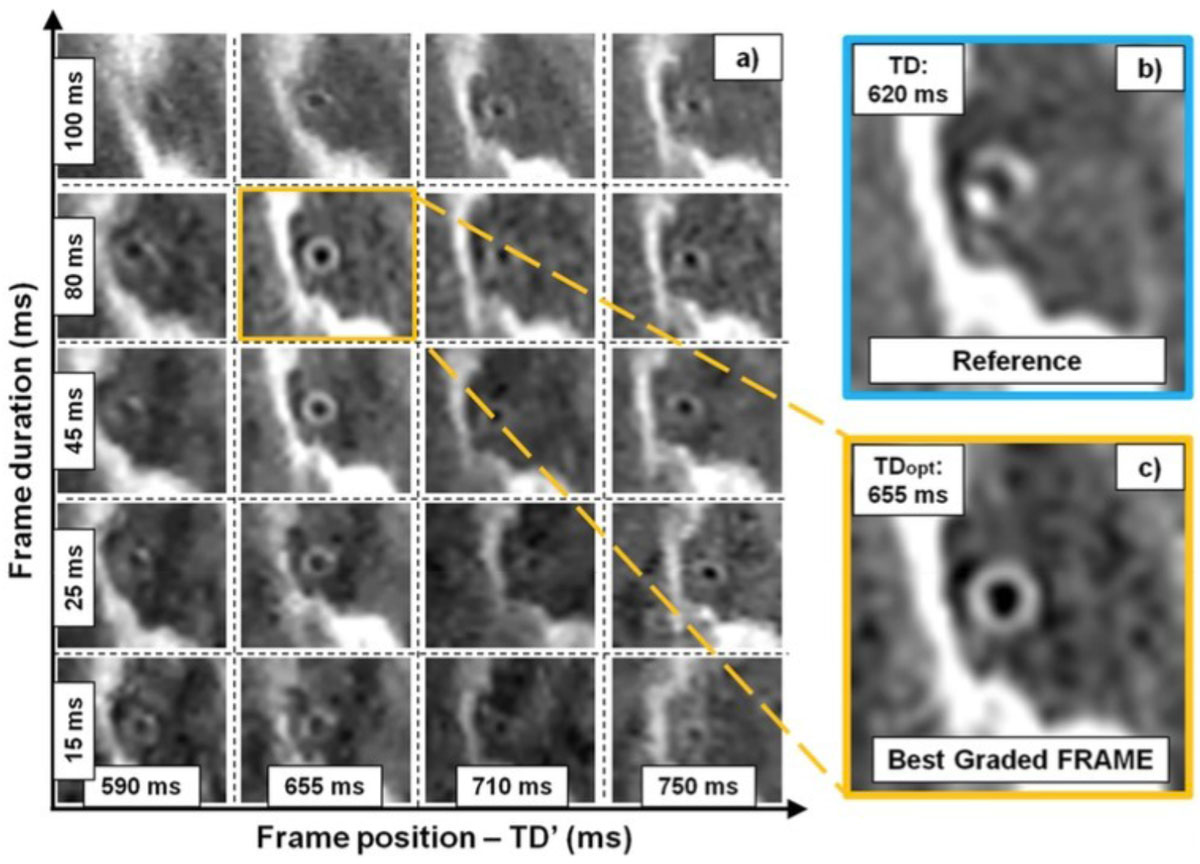
**Reconstructed images in one volunteer**. For each dataset, multiple frames were created by freely choosing their position and duration, as shown in a. Images were then graded and that with the highest grade (orange square, c) was quantitatively compared to the baseline image (blue square, b). A clear improvement in vessel wall delineation between b and c is can be seen. TD of the standard acquisition differed from TDopt of the best graded frame.

Data acquisition was performed at 3T (Prisma, Siemens), in 15 healthy volunteers, with both a conventional radial DIR (baseline) and a version of it which exploits the golden angle radial trajectory and a prolonged acquisition window (Fig 1). Relevant imaging parameters were: flip-angle 16°, FOV 260 mm^2^, TE 3.2 ms, TR 6.5 ms, slice thickness 8 mm, spatial resolution 0.6 mm^2^. The inversion time (TI) and the trigger delay (TD) were prescribed accordingly to the heart rate of the subject. Acquisition windows were 50-110 ms (baseline) and 150-330 ms (prolonged window). During post processing, the prolonged acquisition window was sub-divided into multiple frames, characterized by different position within the cardiac cycle (TD') and temporal width. The frame providing the best vessel wall depiction among the reconstructed ones was compared with the baseline (Fig 2). For both images (baseline and best graded frame), the following end-points were quantified (4); vessel wall thickness (VWT), percentage vessel wall sharpness (VWS%), extent of visible vessel wall circumference (VWC). In addition, two experts were asked to grade images from 0 (bad quality) to 5 (excellent quality). For the best graded frame, its position (TDopt) was stored for further comparisons.

## Results

The quantified vessel wall thickness remained unchanged among the two reconstructions (0.95 ± 0.27 for the baseline, and 0.97 ± 0.18 for the best frame, p = NS). Simultaneously, image quality improved (Fig 2) according to all the quantified end-point from the baseline to the best graded frame (VWS%: 24.2 ± 8.7% and 42.5 ± 7.0%, VWC: 5.6 ± 3.1 mm and 7.2 ± 0.2 mm, visual grading: 2.2 ± 1.0 and 3.4 ± 0.8, p < 0.03 in all cases). Furthermore, TD was found to be different from TDopt (665.7 ± 88.1 ms and 687.3 ± 92.7 ms, p < 0.01).

## Conclusions

We successfully implemented a flexible technique for quantification of coronary vessel wall thickness which eliminates difficulties related to complicate acquisition planning of conventional BB DIR. With this technique, improved vessel wall delineation can be obtained. Patient studies are now warranted.
